# The Synovium Theory: Can Exercise Prevent Knee Osteoarthritis? The Role of “Mechanokines”, A Possible Biological Key

**DOI:** 10.3390/jfmk4010011

**Published:** 2019-01-23

**Authors:** Michelino Di Rosa, Paola Castrogiovanni, Giuseppe Musumeci

**Affiliations:** 1 Department of Biomedical and Biotechnological Sciences, Human Anatomy and Histology Section, University of Catania, 95100 Catania, Italy; 2 School of the Sport of the Italian National Olympic Committee “CONI”, 71368 Sicily, Italy

Osteoarthritis (OA) is a debilitating disease widespread in the world, having a negative impact on daily activities, especially in old age [1,2]. The clinical studies on OA are numerous, with the aim of finding appropriate therapeutic solutions (pharmacological and otherwise) that can make the daily lives of OA patients easier. Even experimental studies are increasingly numerous, and in this case the main purpose is to understand the possible molecular pathways involved in the pathogenesis of OA [1,2]. If you know the problem, it is easier to find solutions. Even if OA is not completely solved by therapeutic treatments, an adequate lifestyle can prevent and, in any case, support the patient, especially by alleviating pain and motor inefficiency, which are evident signs of the disease. Physical activity, as it is known, is a practice that is always advisable in everyone’s life because its beneficial effects are felt in the body at 360°, both at systemic and local levels. Obviously, it should be managed in a coherent way with the achievement of a certain result because the consequences of light, moderate, or vigorous physical activity are very different, and therefore it is always necessary to adapt the physical activity to the individual and to his/her specific physical characteristics [1,2].

Our aim of this editorial is to identify a hypothetical mechanism of action, as a possible biological key, concerning the possible immunological/molecular developments in the OA process, during both static and dynamic conditions, within the cellular constituents of the synovial membrane. 

During OA development, for the hypo-mobility condition (Figure 1A), Type A synoviocytes play a key role in the antigen presentation and starting the local innate immune activation. During this process, we have an alteration of the microenvironment, mediated by the release of inflammatory cytokines (IL-1β, TNF-α), chemokines (IL-8, CHI3L1) [3], and digestive enzymes (MMPs), that mediate tissue damage, inflammation, and immune-cells recruitment (1st phase—state 1 and 2). The digestion of the autologous fragments, coming from the cartilage destruction, activate the Type A synoviocytes. In this context, the exposition of digested material through the Major Histocompatibility Complexes (MHC class I and class II) prepares the Type A synoviocytes to dialogue with the lymphocytes through their T cell receptors (TCR). The local recruitment of naïve T helper lymphocytes (Th0) to the synovium cavity activates the 2nd phase of the immunological response, mediated by the action of acquired immunity (state 3). The Th0 switch to Th1 and, coming into contact with Type A synoviocytes, will polarize the response to an inflammatory state (Type A synoviocyte 1 act as macrophages polarized in M1), mediated by the release of Th1 cytokines such as IFN-γ, TNF-α, and IL-1β [4,5] (states 4 and 5—2nd phase). All these molecules will contribute to exacerbate the matrix degradation. The Th1 lymphocytes also have effects on Type B synoviocytes (synthesis action), turning them into fibroblast-like synoviocytes (FLS) [6] (state 6) with the consequent reduction of the synthesis activity of specialized matrix constituents and synovial fluid, including lubricating molecules such as lubricin and hyaluronan (state 7—3rd phase).

Instead in OA patients, during Moderate Physical Activity (MPA) (Figure 1B), the synovium is subjected to changes in the microenvironment. The flexion and extension exercises stimulate the release of synovial fluid into the joint cavity, and consequently, through the load and shear movements of the synovial fluid, the primary cilia of Type B synoviocytes are also stimulated (state 1). The primary cilium is a non-motile and microtubule-based organelle capable of playing a number of roles in mechano-, chemo-, and osmo-sensitive functions [7,8]. It is a prime candidate for mechano-regulation and it activates the Type B synoviocytes to secrete more specialized matrix constituents, such as lubricin and hyaluronic acid, into the joint cavity to lubricate and nourish the cartilage (state 2). Furthermore, the fluid shear movement also stimulates the differentiation of the synovial mesenchymal stem cells (MSCs) in chondrocytes to form neo-articular cartilage of the superficial layer [9,10] (state 3). The microenvironment modification induced by Type B synoviocytes plays a key action in the recruitment and polarization of Th0. The production of regulatory cytokines such as IL-4, IL-10, and IL-13 by T cells inhibits Type A synoviocytes from producing destructive cytokines (IL-1β and TNF-α) [11]. Moreover, the presence of these cytokines in the microenvironment switches the immunological response from Th0 to Th2 (state 4). As a consequence, Type A synoviocytes will undergo the polarizing event towards a similar macrophage M2 (Type A synoviocytes 2). M2 polarize through the interactions with the anti-inflammatory “mechanokines” IL-4 and IL-10 (state 5). The names of these anti-inflammatory cytokines (IL-4 and IL-10) involved in the mechanobiology conditions could be called “mechanokines”. We would like to coin this term for the first time in the literature. Under this condition, the endogenous release of IL-1β and TNF-α usually decreases, whereas IL-4 and IL-10 increase [12] (state 6). Furthermore, the IL-6 secretion enhances the expression of the naturally occurring tissue inhibitors of MMPs (TIMPs), with the consequence of the reduction in the destruction of the matrix (state 7).

We would like to coin this term “mechanokines” for the first time in the literature, even if the anti-inflammatory cytokines IL-4 and IL-10 could be produced also in those tissues/locations which function is not modulated by mechanical forces.

In conclusion, the benefits of moderate physical activity in OA patients may have protective roles on joints as a non-surgical and non-pharmacological treatment, re-establishing the physiological function of synoviocytes, preventing the onset of OA, and/or postponing the need for joint replacement. To maintain joint health is fundamental for independent living, good health status, and quality of life.

## Figures and Tables

**Figure 1:**
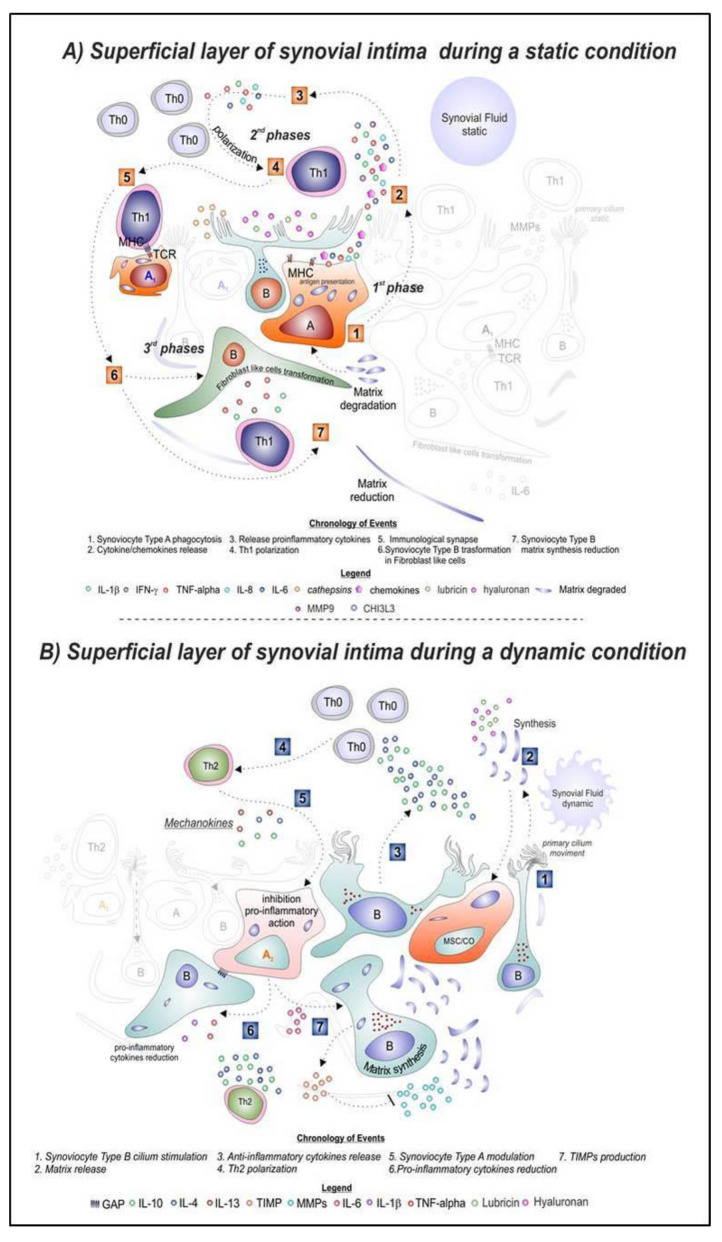
Graphical representation of the superficial layer of synovial intima in osteoarthritis (OA) during a static (**A**) or dynamic (**B**) moderate movement conditions. It is possible to identify the Type A synoviocytes polarized in similar macrophages M1 and M2; Type B synoviocytes; primary cilium; TH0, TH1, and TH2 lymphocytes; and the relative cytokines/chemokines secretion during the two conditions.
